# Multi-omics approaches to major psychiatric disorders

**DOI:** 10.1007/s40211-025-00564-0

**Published:** 2025-12-16

**Authors:** Mojtaba Oraki Kohshour, Alba Navarro-Flores, Urs Heilbronner, Thomas G. Schulze

**Affiliations:** 1 Institute of Psychiatric Phenomics and Genomics (IPPG), LMU University Hospital, LMU Munich, 80336 Munich, Germany; 2 Department of Immunology, Faculty of Medicine, Ahvaz Jundishapur University of Medical Sciences, Ahvaz, Iran; 3 International Max Planck Research School for Translational Psychiatry (IMPRS-TP), Munich, Germany; 4 Department of Psychiatry and Behavioral Sciences, Norton College of Medicine, SUNY Upstate Medical University, Syracuse, NY USA; 5 Department of Psychiatry and Behavioral Sciences, Johns Hopkins University School of Medicine, Baltimore, MD USA

**Keywords:** Schizophrenia, Bipolar disorder, Major depressive disorder, Data integration, Early diagnosis, Schizophrenie, Bipolare Störung, Schwere depressive Störung, Datenintegration, Frühdiagnose

## Abstract

In recent years, major psychiatric disorders have been intensively researched. Studies have investigated the pathophysiology of these disorders in detail and at various molecular levels with several omics techniques, including genomics, epigenomics, transcriptomics, proteomics, and metabolomics. However, although the results of a single omics study can help shed light on some of the unclear aspects of the biological circuits involved in the pathophysiology of major psychiatric disorders, the complexity of the biological mechanisms underlying these conditions makes it necessary to consider multiple types of omics data and multiple levels of analysis, including various conceptional, methodological, and quality control criteria. Currently, dealing with high-dimensional data and sparse heterogeneous data structures remains one of the biggest challenges to integrating data from multi-omics approaches. The hope is that eventually the development and application of methods to integrate biological and phenotypic data through multi-omics and machine learning-based algorithms may allow early diagnosis of major psychiatric disorders, perhaps even before disease onset, and enable accurate, personalized treatment. In this mini-review, we summarized the main findings of the field by reviewing systematic reviews, meta-analyses, and narrative reviews on the major psychiatric disorders schizophrenia, bipolar disorder, and major depressive disorder.

## Abstract (de)

In den letzten Jahren wurden schwere psychiatrische Störungen intensiv erforscht. Studien haben die Pathophysiologie dieser Störungen detailliert und auf verschiedenen molekularen Ebenen mit verschiedenen Omics-Techniken untersucht, darunter Genomik, Epigenomik, Transkriptomik, Proteomik und Metabolomik. Obwohl die Ergebnisse einer einzelnen Omics-Studie dazu beitragen können, einige der unklaren Aspekte der biologischen Kreisläufe, die an der Pathophysiologie schwerer psychiatrischer Erkrankungen beteiligt sind, zu beleuchten, ist es aufgrund der Komplexität der diesen Erkrankungen zugrunde liegenden biologischen Mechanismen wichtig, mehrere Arten von Omics-Daten und mehrere Analyseebenen zu berücksichtigen, darunter verschiedene konzeptionelle, methodische und Qualitätskontrollkriterien. Derzeit stellt der Umgang mit hochdimensionalen Daten und spärlichen heterogenen Datenstrukturen nach wie vor eine der größten Herausforderungen bei der Integration von Daten aus Multi-Omics-Ansätzen dar. Die Hoffnung ist, dass die Entwicklung und Anwendung von Methoden zur Integration biologischer und phänotypischer Daten durch Multi-Omics- und maschinell lernbasierte Algorithmen letztendlich eine frühzeitige Diagnose schwerer psychiatrischer Erkrankungen, möglicherweise sogar vor Ausbruch der Krankheit, sowie eine genaue, personalisierte Behandlung ermöglichen. In diesem Manuskript haben wir die wichtigsten Erkenntnisse auf diesem Gebiet zusammengefasst, indem wir systematische Übersichtsarbeiten, Metaanalysen und narrative Übersichtsarbeiten zu den wichtigsten psychiatrischen Erkrankungen Schizophrenie, bipolare Störung und Major-Depression ausgewertet haben.

## Introduction

Globally, psychiatric disorders are one of the main causes of disability. Furthermore, major psychiatric disorders, in particular schizophrenia (SCZ), bipolar disorder (BD), and major depressive disorder (MDD), remain among the top 10 leading causes of mental health issues [1].

SCZ affects about 1% of adults, and behavioral genetic studies have demonstrated a high heritability (about 80%). It is a severe, heterogeneous, and debilitating neuropsychiatric disorder with a complex biological mechanism [1, 2]. BD produces cycles of depressive and manic episodes and is associated with a high risk of suicide; like SCZ, it is also a complex entity, and many of the underlying pathogenic processes remain unknown [3]. MDD is a heterogeneous, highly prevalent, and burdensome psychiatric disorder. Its diagnosis and therapeutic options remain challenging because of the high interindividual heterogeneity in the clinical and biological correlates of the disorder [4].

Research on psychiatric disorders has always focused on strategies for differential and precise diagnosis and the development of targeted and effective treatments. However, the complicated and multifactorial character of major psychiatric disorders means that decoding their dimensions requires several approaches and methodologies at various molecular levels. To this end, omics research and its potential applications in complex disorders have received much attention in recent years. The past decade has seen the rapid implementation of various omics techniques, such as genomics (i.e., DNA sequence data), transcriptomics (i.e., RNA expression levels), epigenomics (i.e., epigenetic alterations), proteomics (i.e., protein levels), and metabolomics (i.e., metabolite levels), to investigate the pathophysiology of psychiatric disorders in depth at different levels [2]. A key difference to earlier research is that on each biological level, large sets of molecules can be simultaneously studied with high-throughput technologies.

Each omics approach provides insights into the biochemical pathways behind the pathophysiology of psychiatric disorders, but analyzing integrated large-scale data from various omics types (i.e., meaningfully combining multi-omics data to provide a more comprehensive view of a biological phenotype [5]) will result in a more comprehensive understanding of the key players and interactions involved in the various biological mechanisms behind the phenotype of interest, an approach that will greatly aid biomarker discovery and, eventually, efficient clinical translations and personalized medicine [2, 5].

**Fig. 1:**
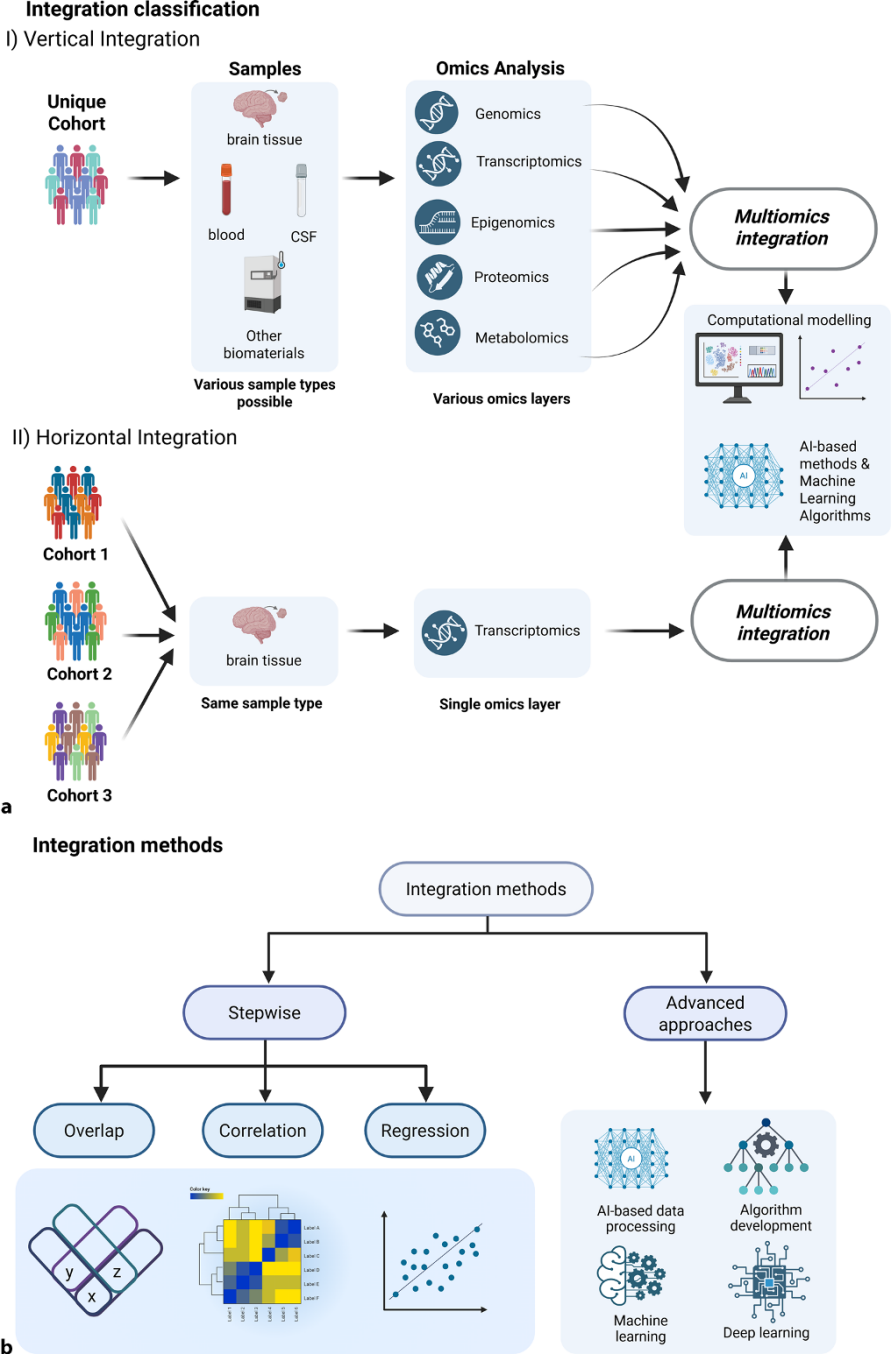
Multi-omics integration classification and methods. **a** Classification of integration methods, I) vertical integration refers to the process of analyzing different omics layers of information from the same participants in a cohort, which could be obtained from various sample types relevant for psychiatry; while in II) horizontal integration, different cohorts provide information from the same omics layer analyzed from the same sample type to be further integrated. **b** Integration methods relevant for psychiatry include the stepwise approach—where data can be integrated by simply overlapping the results, or by correlation or regression—and advanced methods—which by use of artificial intelligence (AI) tools facilitate the development of new algorithms for data analysis or for classification and clustering using machine learning or deep learning methods (*Created with BioRender.com*). *CSF* cerebrospinal fluid

In general, multi-omics data are integrated with two approaches: i) the multistage (stepwise or hierarchical) approach, which analyzes each type of omics data independently and then integrates the results, and ii) the meta-dimensional approach, which analyzes the various types of omics data simultaneously by using advanced strategies, such as concatenation, transformation, and integration of the models (Fig. 1; [5]). In psychiatry, these approaches have been performed with various techniques, such as: i) integrating genomics (i.e., results of genome-wide association studies [GWASs]) with other omics data by using enrichment-based, statistical fine-mapping and imputation-based methods; ii) machine learning (ML) methods for (diagnostic) classification, outcome and risk prediction, and treatment response prediction; and iii) functional enrichment with model systems (human cell lines and animal models) [6].

In recent years, numerous cohorts and large-scale research projects have been performed in the form of brain-specific consortia, such as psychENCODE, CommonMind, and BrainSeq, to coordinate and investigate the integration of multi-omics data on psychiatric disorders [7]. The goals of such integration can be classified into three general categories: i) to subtype and classify psychiatric disorders; ii) to discover diagnostic and therapeutic biomarkers; and iii) to advance our understanding of biological processes [7].

In this mini-review, we present the overall findings of multi-omics studies on three major psychiatric disorders, SCZ, BD, and MDD. Studies were identified by searching for and reviewing systematic reviews, meta-analyses, and narrative review articles and, in the case of BD, also searching for individual studies.

## Methods

To identify relevant articles, we performed a literature search in PubMed for systematic review, meta-analysis, and narrative review articles (as search filters) that directly mentioned the disorders of interest. We included articles on human studies that were published in English by the end of June 2025. Our search used the following strings and resulted in the following numbers of hits:

i) (“Multi-omics” [tiab] OR multiomics[tiab]) AND schizophrenia[tiab]: 27 hits
ii) (“Multi-omics”[tiab] OR multiomics[tiab]) AND (“bipolar disorder”[tiab] OR bipolar[tiab]): 12 hits, and
iii) (“Multi-omics”[tiab] OR multiomics[tiab]) AND (“MDD”[tiab] or “depression”[tiab] OR “major depressive”[tiab]): 35 hits.

After screening the resulting articles, we removed duplicates and publications unrelated to the topic of interest. Of the 27 hits for SCZ, seven studies were eligible for inclusion. Of the 12 hits for BD, only one study was eligible, so we removed the PubMed search filters, i.e., systematic review, meta-analysis, and narrative review, and consequently identified 30 additional studies, three of which were eligible for inclusion. In addition, of the 35 hits for MDD, three studies were eligible for inclusion.

## Results

### Findings of SCZ studies

The primary conclusion drawn from the findings of individual omics studies on SCZ is that the pathophysiology of the disorder could be influenced by the effects of environmental factors on various molecular levels, particularly the dopaminergic, glutamatergic, and inflammatory pathways [2].

Regarding multi-omics approaches, researchers have suggested that integrated omics studies on SCZ can be divided into four perspectives: pathogenesis, classification, risk prediction, and precise intervention [2]. These perspectives have been investigated by a variety of integrative omics models, such as genomics–transcriptomics, genomics–epigenomics (a methylome-wide association study), genomics–metabolomics, and genomics–connectomics (i.e., analyzing genomics and the connections within the brain). For instance, regarding the pathogenesis perspective, a transcriptomic imputation analysis, which combines expression quantitative trait locus reference panels with large-scale genotype data, used gene expression predictors to analyze the dorsolateral prefrontal cortex and other brain regions and identified 413 genome-wide significant associations (including 67 nonmajor histocompatibility complex genes, 14 of which were novel) across 13 brain regions and 36 significantly enriched pathways, including pathways involved in hexosaminidase A deficiency and multiple porphyric disorder [8].

In the classification perspective, another transcriptome imputation study offered an analytical approach that used GWAS-predicted gene expression levels and clinical data to classify individuals with SCZ into three subgroups that showed significant differences in treatment response and prognosis [9].

In relation to the risk prediction perspective of SCZ, an integrative analysis of genotype data (from GWASs) and gene expression/regulation data (from functional genomics) embedded the gene regulatory network into a deep-learning model and consequently improved the accuracy of the SCZ prediction model approximately sixfold compared with the conventional single nucleotide polymorphism (SNP)-based polygenic risk scores (PRS) [10].

Regarding the precise intervention perspective, a transcriptome-wide association study used a pharmacogenomic approach, which links genetic variants to antipsychotics response, to compare the differences between the imputed transcriptome from SCZ GWASs and drug-induced gene expression profiles [11]. The study highlighted a novel framework for medication repositioning (i.e., finding new indications for existing medications) and found that repositioning candidates were enriched for multiple antipsychotics. It also identified some potential new medications for SCZ [11].

In human brain tissue, a concurrent transcriptomics, proteomics, and metabolomics study found abnormalities in these molecular layers that were linked to glucoregulatory responses in SCZ [12]. Other researchers have proposed that in individuals with SCZ, elevated glucose demand within the prefrontal cortex is linked to abnormal glucose profiles, which are accompanied by decreased glycolysis and glycogenesis and increased glycogenolysis [13]. So far, transcriptomic analyses have discovered several alternative splicing processes for some of the SCZ risk genes identified by genomic research; interestingly, most of these processes occur in genes that are important for neurodevelopment, neuroplasticity, and cognition [14]. Because miss-splicing events may be involved in SCZ etiology, identifying alternative splicing mechanisms for SCZ risk genes and determining the functional implications of these genes’ altered messenger RNA (mRNA) expression is essential for translating genetic discoveries into novel therapeutic approaches [14]. A transcriptomics-neuroimaging study on transforming growth factor-beta 1 (TGF-β1; an important cytokine for brain development and regenerative processes) found that individuals with SCZ exhibited higher levels of TGF-β1 at the mRNA and protein levels and a significantly thinner cortex in the lateral occipital region than controls; in line with these findings, the individuals with SCZ showed a negative correlation between TGF-β1 levels and visual cognition [15].

In SCZ, the application of genomics-neuroimaging models offers insight into a possible gene-related pathogenesis from the perspective of changes in brain structure and function. One review paper presented results from several magnetic resonance imaging studies that investigated how distinct SNPs in the coding and noncoding regions of various genes affect the functional and structural connectivity and structure of the brain [16]. The authors mentioned that the trans-scale and multi-omics analysis approach that integrates genomics, connectomics, and radiomics is practical for visualizing the connection between functional genetic variations and the neuroanatomical heterogeneity of SCZ [16]. By integrating genomic, transcriptomics, and neuroimaging data from individuals with SCZ and classifying individuals of two SCZ cohorts into broad neural cell-based subtypes, the authors showed that interindividual variations in cell type-specific functions were associated with changes in cortical thickness [17]. Additionally, by applying SCZ risk alleles enriched in cellular functions, they verified the cell-based classification against the genetic variation unique to each patient [17].

A study that combined data from genomics, diffusion tensor imaging, and resting-state functional magnetic resonance imaging showed that higher PRS for SCZ are linked to disrupted white matter integrity and functional connectivity in individuals with SCZ [18]. An integrative analysis of multi-omics data, i.e., of genomics, serum metabolomics, fecal metagenomics, and neuroimaging data, found that the altered metabolome and dysregulated microbiome were associated with neuroactive metabolites, including gamma-aminobutyric acid (GABA), tryptophan, and short-chain fatty acids [19]. It also found that changes in the GABA and tryptophan neurotransmitter pathways were associated with PRS for SCZ and that GABA perhaps plays a more significant role than the other neuroactive metabolites [19]. Another multi-omics investigation on SCZ that used data from genomics, transcriptomics, neuroimaging, and clinical measurements identified 19 distinct genes that were associated with 16 brain areas [20]. Subtype analyses based on gray matter alterations in the target brain region provided a comprehensive understanding of how genetic variations may impact brain architecture and, thus, result in various disorder manifestations [1, 20].

### Findings of BD studies

One study used a multi-omics approach to examine the microbiota–gut–brain axis in medication-free individuals with BD [21]. The authors found that alterations in the microbiota were linked to changes in neuroactive metabolites such as pantothenic acid, riboflavin, folic acid, pyridoxine, kynurenic acid, GABA, and short-chain fatty acids. Additionally, in functional imaging scans they found differences in connectivity in relevant areas, such as the hippocampus, amygdala, superior temporal gyrus, and sensorimotor gyrus [21].

The single-cell sequencing technique presents advantages for multi-omics approaches because it can capture cell-type specific characteristics and reduce heterogeneity. One study applied the single-cell disease relevance score method to integrate single-cell resolution RNA sequencing and PRS based on embryonic and fetal brain tissue (i.e., the dorsolateral prefrontal cortex, dorsal pallium, and cortical plate) [22]. The authors analyzed four BD-related omics panels, including brain single-cell RNA-seq data, cell transposase-accessible chromatin using sequencing (ATAC-seq) data, bulk-RNA sequencing data, and GWAS and transcriptome-wide association studies (TWAS) data and found a novel cell cluster expressing adenylate cyclase 1 in the human brain. Moreover, they showed that astrocytes, microglia, and oligodendrocyte precursor cells were significantly associated with BD in different analyses and that the BD-associated genes presented particular characteristics in the various omics assessments [22].

A favorable response to lithium—the first-line treatment in BD—helps to reduce the burden of the condition; however, biomarkers related to this treatment phenotype are lacking. A review of various omics studies in this field found that the target genes related to BD show the involvement of the brain-derived neurotrophic factor gene (*BDNF*; a protein coding gene involved in neuronal survival, synaptic transmission, and plasticity) in both BD and lithium response [23]. Although methylation patterns in individuals with BD were contradictory and inconclusive, lithium response was partially associated with epigenetic changes through distinct methylation patterns and changes in related enzymes, whereas microRNA alterations remained a promising explanation that still requires further research.

A genomics–transcriptomics study in individuals with BD used data from induced pluripotent stem cell-derived neurons and GWASs on lithium response in responders and nonresponders to obtain candidates related to lithium response phenotype. A total of 1119 candidates were selected, and gene enrichment analysis showed that processes related to the extracellular matrix and focal adhesion were the most relevant [3]. The authors hypothesized that in lithium responders, the extracellular matrix shows defects that impact focal adhesion and are therefore the pathophysiological cause of BD. In these individuals, lithium helps revert the defects and reverse the symptoms of BD, which explains these individuals’ favorable response to the drug [3]. Finally, pharmacometabolomics studies showed that individuals with BD present altered lipid metabolites in the brain and plasma [23].

### Findings of MDD studies

A review article on mood disorders considered eight studies that used multi-omics models in individuals with major depression [24]. In these studies, the data integration strategies utilized included the step-by-step strategy, which detects only strong variance, and the advanced strategy, which makes use of ML/deep learning methods (Fig. 1b).

The step-by-step strategy includes overlapping, correlation, and regression approaches (Fig. 1b). One study used the overlapping approach to compare response to escitalopram in responders, nonresponders, and healthy controls and found 16 differentially methylated CG sites in relevant genes related to neurodevelopmental hippocampal axon pruning and synaptic plasticity [25]. Another study also used this approach to analyze mRNA and DNA methylation in individuals with MDD and controls; it showed that the overlapped hypomethylated and upregulated genes were related to phosphatidylinositol 3‑kinase/protein kinase B (PI3K-Akt), interleukin 17, and axon guidance signaling pathways, whereas the overlapped hypermethylated and downregulated genes were related to the mitogen-activated protein kinase (MAPK), and nuclear factor k-light-chain-enhancer of activated B cells (NF-kB) signaling pathways [26]. The correlation approach was used in a GWAS in twins discordant for MDD, and the study found overrepresentation of differentially methylated genes in the individuals with MDD [27]. Another study used the regression approach to understand the mRNA, microRNA, and clinical correlates of treatment-worsening suicidal ideation and found that a logistic model including miR-5695, STMN1 mRNA, and the Montgomery–Asberg Depression Rating Scale score at baseline predicted this outcome [28].

The advanced strategy was used by two studies. The first integrated data from GWAS and data on expression quantitative trait locus, RNA-seq in the dorsolateral prefrontal cortex, and chromatin conformation (Hi-C) from large cohorts of individuals with MDD and found two implicated genes related to (i) synapse formation and differentiation (*LRFN5*) and (ii) synaptic plasticity (*DCC*) [29]. The second study used differentially methylated CpG sites to develop a random forest classifier to detect suicidal behavior and showed an accuracy of 92.6% [30].

The diagnosis of depression remains a challenge because of the lack of valid biomarkers. Aiming to identify such biomarkers, a scoping review evaluated studies that applied ML models to omics data for clustering individuals with depression [4]. The article included 15 studies that used various omics types, such as genomics, epigenomics, transcriptomics, and microbiomics, to evaluate samples from blood, brain, and stool. Among these studies, only one integrated multi-omics data by using a random forest classifier to accurately predict suicidal behavior (see above [30]). Another study used ML analysis of microRNA expression data to differentiate between medication-free individuals with MDD and controls and showed high accuracy of the approach (AUC = 0.97) [31]. Moreover, the authors were able to separate individuals with mild MDD from those with moderate or severe MDD by using unsupervised ML in two clusters of patients (AUCs of 70 and 76).

Antidepressant treatment represents another challenge because of the high interindividual heterogeneity in dose response and tolerance. In the case of treatment-resistant depression, multi-omics approaches have revealed an association with alterations related to i) the immune system, inflammatory pathways, and the hypothalamic–pituitary–adrenal (HPA) axis; ii) neuroplasticity; iii) calcium signaling; iv) neurotransmitters; and v) other factors, such as the cytoskeleton, blood coagulation, and apoptosis/autophagy. These findings were supported by genomics, transcriptomics, and metabolomics data, among others [32].

## Conclusion and future perspectives

The complexity of psychiatric disorders, particularly their heterogeneity and biological variability, especially in the brain, means that more sophisticated approaches and techniques for analyzing large-scale data are required to shed light on the biological mechanisms involved and, thus, achieve the ultimate goal of improving diagnostic criteria and precise treatment. Initially, individual omics studies helped to improve our understanding of the various molecular layers involved in the biological mechanisms and pathways of psychiatric disorders. Subsequently, integrating data from the various omics disciplines yielded comprehensive insights into the causal relationships between genes, environment, and phenotypes. Accordingly, genomics, which looks for genetic variations at the most basic molecular level, has always been the primary driving force for research, guiding the way for integrating other omics data to facilitate translational medicine [2]. Therefore, as the most fundamental omics type, genomics is included in the majority (> 75%) of integration modalities and can serve as a foundation for analyzing the impact of genomics on other omics types [2, 33]. For example, an integrated analysis of data from genomics and transcriptomics yielded more biologically meaningful insights than separate analyses of the two omics approaches because it identified how the SNPs affect gene expression; combining information from genomics and proteomics elucidated the functional role of SNPs; and studies that integrated genomics and epigenomics emphasized how SNPs alter functions by modulating chromatin accessibility [2].

High-dimensional data from multi-omics approaches help to identify biologically based subtype classifications of psychiatric disorders [2]. In this regard, current research on major psychiatric disorders most commonly uses a combination of functional genomics approaches, such as genomics, epigenomics, and transcriptomics. For instance, a potential therapeutic strategy in SCZ, the altered alternative splicing characterization of SCZ risk genes identified by genomic studies may aid in applying novel approaches to target the spliced mRNAs or proteins of SCZ-associated isoforms [14]. Research has provided evidence that the composition of genetic profiles and gut microbiota are related [2]. Additionally, metabolomics—either by itself or in combination with microbiomics—may aid in the early diagnosis of major psychiatric disorders [2].

The majority of omics data are obtained from whole blood or tissues [5]. However, the use of single-cell omics techniques, which provide omics data from individual cells (e.g., from brain tissue), and the subsequent integration of these data could support useful cell-based diagnostic applications and help to identify therapeutic interventions as a promising strategy for better understanding the impact of cell-based omics types [5, 7, 17]. One of the most significant challenges to integrating various types of multi-omics data is working with high-dimensional data and sparse heterogeneous data structures (i.e., fundamental differences between the various omics datasets being integrated), which makes it difficult to handle the integration of multiple omics levels and data modalities from methodologically different studies and to interpret the results. Further hurdles are related to the unavailability of large and well-characterized cohorts and to performing statistical and computational analyses, obtaining biologically interpretable results, and evaluating output models on the basis of biological processes [6].

Overall, similar to multi-omics data in other fields, the results of various multi-omics studies on major psychiatric disorders still need to be replicated so that they can be applied with more confidence in the next steps toward translational medicine. In this regard, more research and advanced methods are required to pinpoint the precise effects of the identified molecular changes and determine how to apply the findings. Although any type of omics study can help illuminate some of the unclear aspects of the biological mechanisms that underlie the pathophysiology of major psychiatric disorders, the intricacy of the disorders and the need to maximize the effectiveness of the studies to meet the intended objectives make it important to consider several factors, such as the type and quality of samples and data; deep phenotyping and categorization of study participants and research principles (e.g., that consider different environmental factors) based on hierarchically and dimensionally measured criteria (e.g., Hierarchical Taxonomy of Psychopathology [HiTOP], and NIMH Research Domain Criteria [RdoC]); use of measurement tools with satisfactory psychiatric criteria; and the careful combination and analysis of the various omics data, which can help to accurately reveal the biological pathways involved in the pathophysiology of the disorders. The use of advanced statistics and artificial intelligence-based models and ML algorithms designed for large-scale data has the great potential to improve our ability to analyze complex datasets generated by multi-omics studies, making it easier to identify main players and effective mechanisms than with conventional analysis techniques [33]. In the near future, multi-omics and artificial intelligence-driven multimodal technologies (that combine multiple specialized models to process multiple data types for enhanced task performance), together with greater adoption and advancement of multi-omics approaches, could work towards the appropriate combination of genotype and phenotype data and, thus, facilitate early diagnosis and precise, personalized treatment [33].
